# Glutamatergic Neurotransmission Disruption as a Pathomechanism of Brain Damage in Disorders of Amino Acid and Organic Acid Metabolism: Insights from Animal and Cellular Studies

**DOI:** 10.1007/s11064-026-04722-w

**Published:** 2026-03-14

**Authors:** Manuela Bianchin Marcuzzo, Maria Paula Dalla Vechia Benati, Diogo Onofre Souza, Moacir Wajner, Guilhian Leipnitz

**Affiliations:** 1 Programa de Pós-Graduação em Ciências Biológicas: Bioquímica, Universidade Federal do Rio Grande do Sul, Rua Ramiro Barcelos, 2600-Anexo, Porto Alegre, RS 90035-003 Brazil; 2 Programa de Pós-Graduação em Neurociências, Universidade Federal do Rio Grande do Sul, Rua Ramiro Barcelos, 2600-Anexo, Porto Alegre, RS 90035-003 Brazil; 3 Departamento de Bioquímica, Instituto de Ciências Básicas da Saúde, Universidade Federal do Rio Grande do Sul, Rua Ramiro Barcelos, 2600-Anexo, Porto Alegre, RS 90035-003 Brazil; 4 Serviço de Genética Médica, Hospital de Clínicas de Porto Alegre, Rua Ramiro Barcelos, 2350, Porto Alegre, RS 90035-903 Brazil; 5 Programa de Pós-Graduação em Ciências Biológicas: Fisiologia, Universidade Federal do Rio Grande do Sul, Rua Ramiro Barcelos, 2600-Anexo, Porto Alegre, RS 90035-003 Brazil

**Keywords:** Inborn errors of metabolism, Convulsions, Glutamatergic neurotransmission, Excitotoxicity, Animal models

## Abstract

Inborn errors of metabolism (IEMs) are inherited disorders biochemically characterized by the accumulation of potentially toxic metabolites in tissues and body fluids of the affected patients. Although clinical findings are heterogeneous, neurological symptoms, including coma and seizures associated with brain abnormalities, are very frequent. The mechanisms of neurotoxicity of the accumulated metabolites and their effects on cellular functions are still poorly established in many of these disorders. However, animal and cellular studies have shown that disturbances in glutamatergic neurotransmission, potentially leading to excitotoxicity, may represent a relevant mechanism of brain damage in some of these disorders. In agreement with this, treatments for some IEMs aim to mitigate overstimulation of N-methyl-D-aspartate (NMDA) receptors by NMDA receptor antagonists and to reduce the levels of the accumulated metabolites that activate these receptors. This review will focus on pre-clinical data showing disturbed glutamatergic neurotransmission in cells and animal models of IEMs that may offer perspectives for the development of novel adjuvant treatments for these diseases.

## Inborn Errors of Metabolism (IEM)

Inborn errors of metabolism (IEMs) are genetic diseases caused by altered or deficient proteins, particularly enzymes, leading to a blockage in a metabolic pathway and subsequent accumulation of potentially toxic substrates and/or depletion of essential products. IEMs are usually caused by functional mutations in single genes, although a few are caused by activating mutations or structural genomic changes (e.g., in the mitochondrial genome) [1, 2]. Substrate accumulation can lead to the synthesis of other metabolic intermediates through normally inactive enzymatic reactions [3, 4].

IEMs can affect any type of cell or organ and impact any age group from the fetal period to adulthood. Clinical presentation is therefore heterogeneous with nonspecific symptomatology, although involvement of the central nervous system is most frequently observed. Nonspecific symptoms include malnutrition, vomiting, failure to thrive, respiratory distress, hypotonia, seizures, lethargy or coma. It has been postulated that these clinical features may arise from the accumulation of potentially toxic substrates, particularly in the so-called intoxicating IEMs, or alternatively due to product deficiency [5]. Generalized convulsions are predominant in some of these disorders, indicating that excitotoxicity may play a role in the cerebral manifestations [6–8].

IEMs have been classified in various groups based on their pathophysiological mechanisms, clinical manifestations, substrate size and altered biochemical processes. Recently, Ferreira and colleagues updated the term IEMs to inherited metabolic disorders, and established the International Consensus Classification of Inherited Metabolic Disorders (ICIMD) [4]. Thus, more than 1,400 IEMs are currently recognized and catalogued in 24 categories, comprising more than 100 groups. These categories were based on the metabolic pathways that often share pathogenetic mechanisms, have similar clinical presentations, and are often recognized by the same diagnostic methodology. Examples of these categories include organic acidurias, and disorders of the metabolism of fatty acids and ketone bodies, branched-chain amino acids, glycine and serine, and energy substrates [4].

Diagnosis is fundamentally based on laboratorial findings, whose tests are oriented by the clinical features. Genetic analysis is highly recommended for a definitive diagnosis in some cases. Neonatal screening is essential for early diagnosis and prevention of severe symptomatology. In this particular, the utilization of Tandem mass spectrometry increased the number of diseases detected neonatally and helped to predict the time of onset and outcome of the affected patients. However, diagnosis confirmation in the second-tier tests is necessary for most of these disorders, although subject to limitations in terms of time and cost.

Treatment is limited for most IEMs and is often based on reducing the accumulation of toxic metabolites through substrate restricted diets, cofactor supplementation, specific therapies to facilitate urinary excretion and enzyme replacement. However, despite the implementation of early treatment, repeated crises of metabolic decompensation may still occur in many of these disorders and provoke severe and life-threatening symptoms. Liver transplantation or combined liver and kidney transplantation is an option for some IEMs, especially for organic acidurias such as methylmalonic acidemia and maple syrup urine disease (MSUD). However, it does not avoid long-term neurological manifestations [9–12].

## Glutamatergic Neurotransmission

**Fig. 1:**
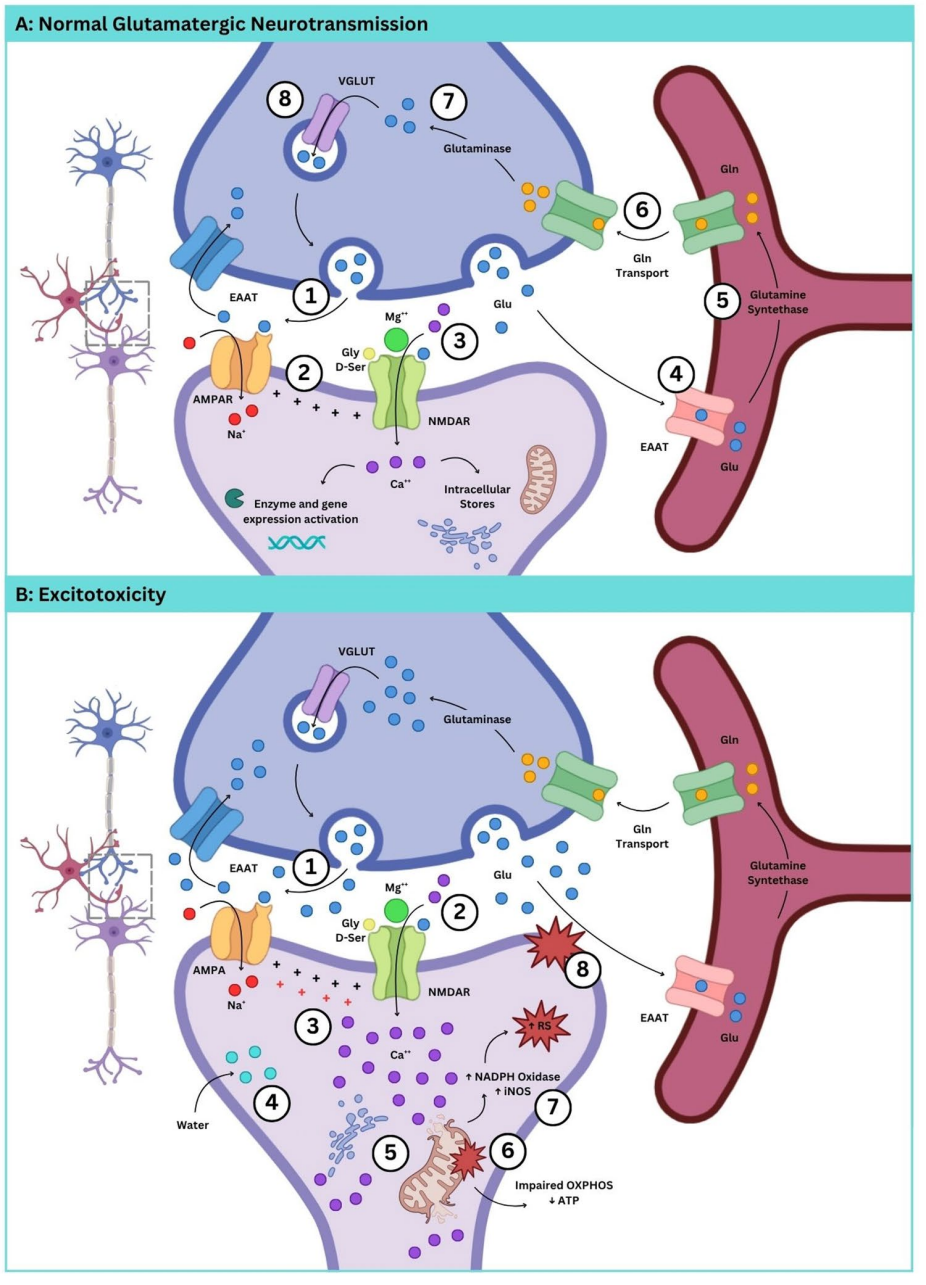
Glutamatergic neurotransmission and excitotoxicity. *Panel A* shows normal glutamatergic neurotransmission with focus on NMDA and AMPA receptors. Under physiological conditions, glutamate (Glu) is released into the synaptic cleft, activating AMPA receptors (AMPAR) (1) and inducing membrane depolarization (2). This depolarization removes the Mg²⁺ block from NMDA receptors (NMDARs) (3), allowing their activation by Glu and subsequent calcium influx. Glycine or D-serine also binds to NMDAR as a co-agonist. (4) Excitatory amino acid transporters (EAATs), abundantly expressed in astrocytes, uptake extracellular Glu. Astrocytes play a crucial role in maintaining glutamatergic homeostasis by converting Glu into glutamine (Gln) (5), which is then shuttled back to neurons to sustain neurotransmitter supply (6). Gln is converted back to Glu within the neuron (7) and stored in synaptic vesicles for subsequent release during neurotransmission (8). *Panel B* depicts glutamate excitotoxicity. In pathophysiological conditions, excessive amounts of glutamate (Glu) are released into the synaptic cleft, leading to the overstimulation of Glu receptors (1). This causes an excessive influx of extracellular Ca²⁺ through overactivated NMDARs (2), resulting in membrane depolarization loss (3). The massive Ca²⁺ entry is accompanied by osmotic water diffusion, causing neuronal swelling (4). Elevated cytosolic Ca²⁺ levels also trigger its release from intracellular stores, which become unable to buffer the excess calcium (5), further increasing cytosolic Ca²⁺ levels. High influx of Ca²⁺ into the mitochondria may open the permeability transition pore, which causes the loss of mitochondrial membrane potential and impairs oxidative phosphorylation, thereby reducing ATP production (6). These events contribute to the activation of NADPH oxidase and nitric oxide synthase (iNOS), promoting excessive production of reactive species (RS) and the activation of phospholipases that degrade neuronal membrane phospholipids (8), among other deleterious effects. Created with Canvas.com

L-Glutamate (Glu) is one of the most abundant amino acids in the CNS [13] and the main excitatory neurotransmitter, playing a crucial role in neuronal development and synaptic plasticity [14]. Glu acts on two main classes of receptors: ionotropic receptors (iGluRs) and metabotropic G protein-coupled receptors (mGluRs). Upon Glu binding, iGluRs allow the influx of Na^+^, K^+^ and Ca^2+^, mediating the excitatory neurotransmission. Subsequently, processes related to synaptic plasticity, such as long-term potentiation (LTP) and long-term depression (LTD), are activated. After exerting its effect, Glu is removed from the synaptic cleft by excitatory amino acid transporters (EAATs) to terminate synaptic transmission [15]. To date, three groups of iGluRs classified according to the affinity of their specific agonists are known: N-methyl-D-aspartate (NMDA), α-amino-3-hydroxy-5-methyl-4-isoxazole (AMPA), and kainic acid receptors [16] (Fig. 1A). These receptors are composed of four subunits, assembled into homomers or heterotetramers, forming a central channel permeable to cations [17].

The NMDA receptor (NMDAR) has greater permeability to Ca^2+^. At rest, the channel is blocked by Mg^2+^. AMPA receptors are assembled from the four subunits GluA1 to GluA4. The subunit composition of a receptor influences its functional properties, particularly its ion permeability and gating kinetics. The GluA2 subunit is especially significant in determining ion permeability since the lack of this subunit renders the receptor calcium permeable [18]. Activation of the AMPA receptor (AMPAR) is necessary to induce membrane depolarization, which is responsible for removing Mg^2+^-mediated inhibition of the NMDAR channel. NMDARs are formed by different combinations of three major subunits: GluN1, GluN2 (GluN2A-D) and GluN3 (GluN3A-B) [19]. Obligate dimers of GluN1 and generally GluN2 shape the traditional heterotetrameric NMDAR and are responsible for the complete activation of NMDARs, whereas GluN3-containing NMDARs elicit the negative regulation of synapse maturation [20–22].

NMDARs that are comprised of the GluN1 and GluN2 subunits require a co-agonist, which can be either glycine or D-serine. While Glu binds to the GluN2 subunit of the NMDAR, the co-agonist binds to the GluN1 subunit [17]. Recent studies revealed that some NMDARs are exclusively controlled by glycine, such as GluN1/GluN3A NMDARs, which are functionally expressed in neurons. Thus, iGluRs may exist as glutamatergic, glycinergic (or D-serinergic), or as hybrids requiring both ligands [23, 24].

Other important modulators of iGluRs, particularly NMDAR and AMPAR, are the neurosteroids, such as cholesterol and neurosteroid pregnenolone sulfate. These molecules have been shown to influence calcium influx, synaptic efficacy, and plasticity through receptor binding [25, 26]. They also mediate indirect effects on Glu homeostasis, including the regulation of presynaptic Glu release and astrocytic uptake, thereby playing a critical role in neuron-glia interactions and in the maintenance of excitatory-inhibitory balance [27, 28].

Kainic acid receptor (GluK) is a cation channel that, depending on the subunit composition, differs in affinity and potency of agonists as well as in functional properties. Five GluK subunits (GluK1-5) have been identified that can form either homomeric or heteromeric functional ion channels [29]. While GluK3 receptors show significantly lower activation sensitivity for Glu, the GluK4 and GluK5 subunits present high affinity but only form functional channels in heteromeric assembly with one of the GluK1-3 subunits [30].

mGluRs are typical G-protein-coupled receptors. So far, eight mGluR subtypes have been identified and categorized into three main groups (group I, II, and III) based on sequence homology, G-protein coupling specificity, and pharmacological properties [31, 32]. Group I mGluRs (mGluR1 and mGluR5) are predominantly coupled with Gαq/11 proteins, which subsequently activate the β isoform of phospholipase C (PLCβ), leading to intracellular Ca^2+^ mobilization and protein kinase C (PKC) activation. Group II (mGluR2 and mGluR3) and group III (mGluR4, mGluR6, mGluR7, and mGluR8) are predominantly coupled to Gαi/o proteins, whose activation inhibits adenylyl cyclase [31, 32]. Notably, mGluR3 is modulated by the neurotransmitter N-acetylaspartylglutamate (NAAG), which triggers Gi/o signaling pathways that inhibit Glu release in response to increased neuronal activity [33, 34]. Additionally, mGluR activation may trigger G-protein-independent signaling mechanisms that regulate ion channels and other downstream signaling proteins [35]. In this particular, group I mGluRs also enhance Glu NMDAR activation [36].

mGluRs are involved in a wide array of functions in the CNS, including mediation of slow excitatory and inhibitory responses, regulation of calcium, potassium and non-selective cation channels, modulation of inflammatory response, inhibition and facilitation of transmitter release, induction of long-term potentiation and depression, and formation of various types of memory [37]. While the activation of group I mGluRs often leads to cell depolarization and increases neuronal excitability, group II and III inhibit neurotransmitter release [38]. Also importantly, activation of mGluRs triggers pro-survival and proliferation pathways such as PI3K/Akt, PKC, and MAPKs [39–41]. For example, mGluR1/5 stimulation was shown to activate PI3K/Akt and extracellular signal-regulated kinase (ERK) pathways, which promote hippocampal long-term depression and prevent neuronal apoptosis, respectively [40, 42].

## Excitotoxicity

Excitotoxicity is a process involving extracellular accumulation of high concentrations of Glu or other excitatory amino acids, causing excessive stimulation of Glu receptors and leading to a characteristic loss of postsynaptic structures, including dendrites and cell bodies. Increased Glu release in the synaptic cleft, abnormal Glu reuptake due to impaired EAAT1 and EAAT2 function, as well as reduced levels of NAAG, are events that may trigger excitotoxicity [34, 43]. Excessive glutamatergic input can further contribute to the excitotoxic event [44]. Excitotoxicity has been frequently observed in many neurological diseases such as epileptic seizures, ischemia and traumatic brain injury, as well as in Huntington’s, Alzheimer’s, and Parkinson’s diseases, multiple sclerosis, and amyotrophic lateral sclerosis [44, 45].

Glu excitotoxicity results in excessive influx of extracellular Ca^2+^ through the overstimulation of NMDAR, which may cause the release of Ca^2+^ from intracellular stores, further increasing the concentration of cytosolic free Ca^2+^ [46]. Noteworthy, the influx of Ca^2+^ regulates membrane excitability and the intensity of synaptic transmission through the activation of intracellular signaling cascades dependent on this ion. Furthermore, this influx is accompanied by the diffusion of water to counterbalance the osmotic pressure, causing swelling of neurons (Fig. 1B) [21].

Among the processes activated by excitotoxicity that can cause neuronal death, the following stand out: changes in oxidative phosphorylation, causing a decrease in ATP production by mitochondria; activation of phospholipases, responsible for degrading phospholipids in the neural membrane; activation of proteases which damage cell structure; activation of NADPH oxidase and nitric oxide synthase (NOS) with consequent overproduction of superoxide and nitric oxide, respectively, which can further increase the production of free radicals (Fig. 1B) [47].

Noteworthy, glutamatergic neurotransmission disturbances have been also revealed in several IEMs. In the following sections, we present several studies displaying excitotoxic damage in animal and cellular models of IMDs. Examples fall into three categories: (1) in vivo models consisting of the administration of IEMs accumulating metabolites to mimic the defect in wild-type rats; (2) in vitro effects of accumulating metabolites in different cell preparations from rodent brain; and (3) knock out (KO) models developed in mice. The IEMs involving excitotoxicity are organized in the following topics according to the groups established by the ICIMD [4].

## Disorders of Amino Acid Metabolism

### Organic Acidurias

#### Glutaric Acidemia Type 1

Glutaric acidemia type 1 (GA1) is a neurometabolic disorder caused by deficient activity of GCDH, which is involved in the degradation of lysine, hydroxylysine, and tryptophan. GA1 is characterized by the predominant accumulation of glutaric (GA) and 3-hydroxyglutaric (3OHGA) acids in the tissues and body fluids. Affected individuals present with acute encephalopathy, hypotonia, and seizures associated with acute loss of motor skills and dystonia, rigidity, and spasticity [48–50]. During acute encephalopathic crises, bilateral degeneration of the caudate and putamen occurs, accompanied by a severe loss of neurons and gliosis. Neuroimaging shows frontotemporal hypoplasia at birth and subependymal nodules along development. Delayed myelination with progressive widening of anterior temporal and Sylvian CSF spaces, as well as alterations of the globus pallidus, substantia nigra, dentate nucleus, thalamus and central tegmental tract are also common [8, 49, 51–53]. Similar findings showing intensive white matter lesions, reactive astrocytes, and striatum neuronal loss were also detected in the genetic mouse model of GA1 (*Gcdh*^*-/-*^) fed with high dietary lysine or protein, which causes a marked accumulation of GA and 3OHGA [54].

**Fig. 2:**
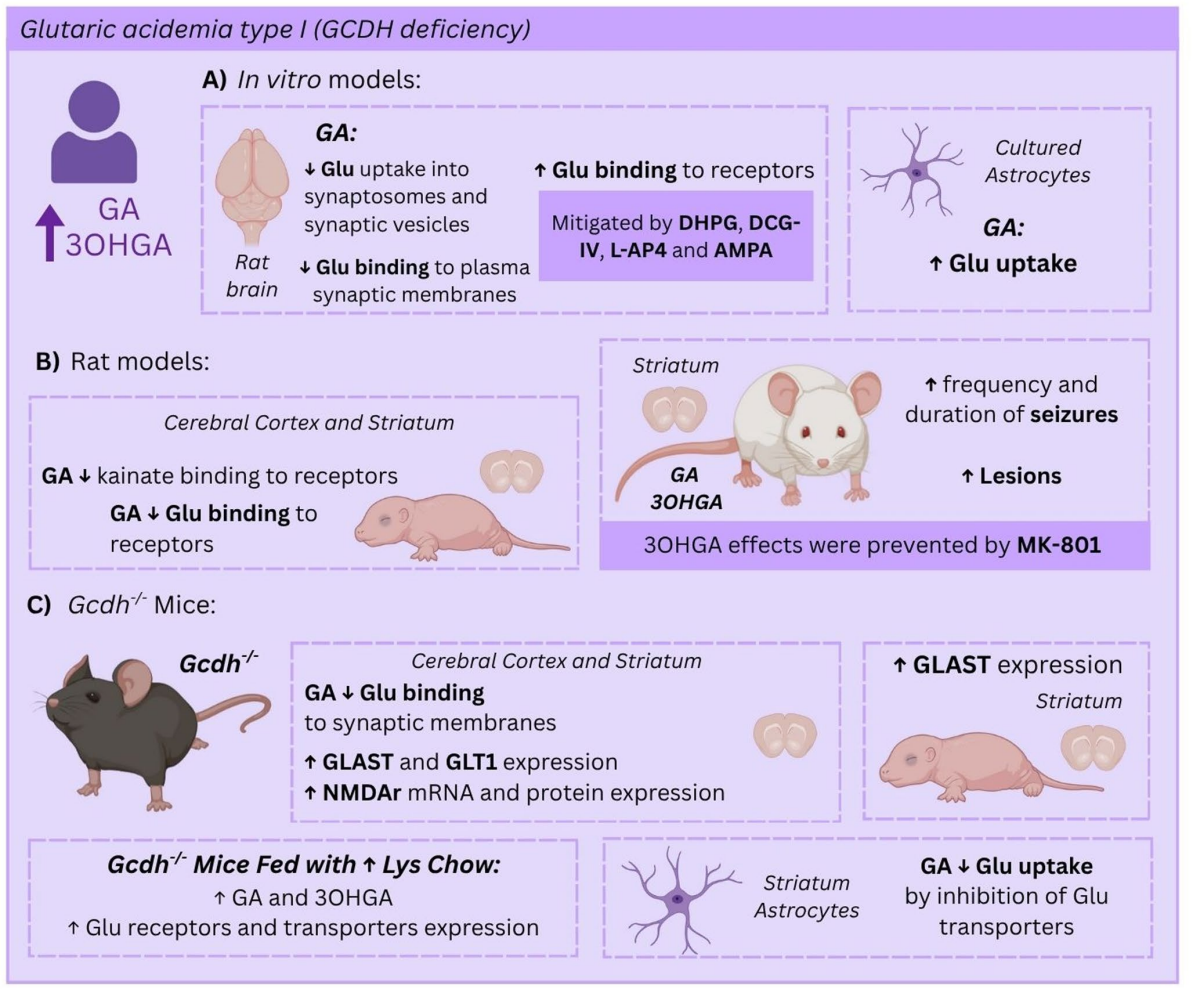
Glutamatergic neurotransmission alterations in glutaric acidemia type I (GA1). (**A**) Glutaric acid (GA) impairs glutamate (Glu) binding to synaptic plasma membranes (receptors), whereas 3-hydroxyglutaric acid (3OHGA) increases seizure frequency and duration, and brain lesion size in rats (**B**). Disturbances in Glu binding and uptake as well as increased gene expression of NMDA receptor subunits and Glu transporters were observed in the brain of *Gcdh*^*-/-*^ mice (**C**). In in vitro rat models, GA impairs Glu uptake and binding and decreases kainate binding in rat brain. NMDAR antagonists mitigate alterations of Glu uptake. Created with Canvas.com

A great deal of animal experimental studies has shown glutamatergic system alterations possibly leading to excitotoxicity in GA1. A scheme of the main effects on the glutamatergic system observed in GA1 is shown in Fig. 2.

 In vitro findings demonstrated that GA reduced Glu uptake into synaptosomal preparations, as well as Glu binding to its transporters in synaptic membranes, potentially leading to excessive amounts of this neurotransmitter in the synaptic cleft [54]. Another report revealed that GA stimulated Glu binding to its receptors in brain synaptic membranes [55]. The same study found that this effect was mitigated by the metabotropic glutamate ligands DHPG, DCG-IV and L-AP4, and by the ionotropic non-NMDA glutamate receptor agonist AMPA, but not by the NMDA antagonist MK-801. Moreover, it was shown that GA inhibited Glu uptake into synaptic vesicles, suggesting alterations in Glu vesicular storage, and increased Glu uptake by astrocytic transporters, implying a disturbance of the glutamine glutamate cycle [56].

Another study showed that GA causes age-dependent differences in Glu binding in rat brain. In this particular, GA reduced Na^+^-independent Glu binding to synaptic plasma membranes (receptors) from cerebral cortex and striatum only in infant (aged 7 and 15 days), but not in adult rats (60 days old). These differential effects were possibly due to organ-specific differences in the expression of Glu receptors and transporters [55]. GA also inhibits kainate binding present in synaptic plasma membranes, indicating that GA binds to kainate receptor [56].

Additionally, intrastriatal administration of GA and 3OHGA to rats caused increased frequency and duration of seizures, indicating in vivo excitotoxic effects [57, 58]. Furthermore, seizure induction promoted by 3OHGA was prevented by MK-801, implying the involvement of NMDAR [57].

Other works using the genetic mouse model of GA1 (*Gcdh*^*-/-*^ mice) also revealed that GA inhibits Na^+^-dependent Glu binding to synaptic membranes (transporter binding) and Glu uptake into the striatum of *Gcdh*^*-/-*^ mice by inhibition of specific astrocyte Glu transporters. High mRNA and protein expression of various NMDAR subunits, and more particularly the subtypes NR2A and NR2B, was observed in the striatum and cerebral cortex of developing *Gcdh*^*-/-*^ mice. Elevated expression of the glutamate transporter subunits GLAST and GLT1 was also found in the cerebral cortex and striatum of adult *Gcdh*^*-/-*^ mice, whereas in infant mice only GLAST mRNA levels were augmented in the striatum. Noteworthy, when exposing these *Gcdh*^*-/-*^ animals to high Lys chow, a further increase in the expression of these glutamate receptors and transporters occurred, implying the participation of the accumulating metabolites GA and 3OHGA in these effects. A comprehensive review of these findings in *Gcdh*^*-/-*^ mice has been published [59].

#### Methylmalonic and Propionic Acidurias

Methylmalonic acidemia (MMAemia) and propionic acidemia (PAemia) are organic acidurias caused by the absent or severe deficient activity of L-methylmalonyl-CoA mutase and propionyl-CoA carboxylase, respectively. MMAemia patients have a characteristic accumulation of methylmalonic acid (MMA), whereas PAemia is biochemically characterized by increased concentrations of propionic acid (PA). MMA can reach concentrations of 2.5–5.5 mM in the blood and cerebrospinal fluid of patients, whereas PA reaches concentrations of 5 mM or higher during metabolic crises [8].

MMAemia and PAemia share similar clinical manifestations, with the most striking features being neurological dysfunction associated with seizures, particularly during metabolic decompensation, as well as developmental delay and intellectual disability. Neuroimaging often reveals alterations in the basal ganglia, white matter abnormalities and cortical atrophy [8, 60]. Treatment consists of protein restriction, a commercial formula without the precursor amino acids forming MMA and PA, L-carnitine supplementation, as well as cobalamin supplementation to responsive MMAemia patients [61, 62].

Disturbances in glutamatergic neurotransmission were shown to be induced by MMA and PA in different animal models. An in vitro study revealed that MMA and PA increased the phosphorylation of cytoskeletal proteins in the cerebral cortex of rats, which was prevented by the NMDAR antagonist DL-AP5. However, CNQX, a non-NMDAR antagonist, and MCPG, a specific metabotropic antagonist, did not alter these effects [63]. Another report demonstrated that MMA increased K^+^-induced Glu release from synaptosomes and reduced Glu incorporation into vesicles [64].

Further data demonstrated that co-incubation of quinolinic acid (QUIN) with MMA or PA elicited NMDAR-mediated synergistic toxic effects in rat brain [65]. Thus, it was found that subtoxic concentrations of MMA or PA (0.5 mM) increased lipid peroxidation in rat brain synaptosomes but did not alter mitochondrial function and ROS production [65]. However, the combination of QUIN with 0.5 mM MMA or PA induced mitochondrial dysfunction and ROS generation, and further increased lipid damage. Furthermore, the NMDAR antagonist kynurenic acid produced in the kynurenine pathway prevented these effects [65]. It is stressed that QUIN is an excitotoxic metabolite of the kynurenine pathway, which is activated during neuroinflammation and is involved in the pathogenic processes of some neurological diseases [66, 67]. A further report demonstrated that MMA-induced lipid peroxidation in rat brain synaptosomes was prevented by MK-801 and antioxidants, including Trolox (hydrophilic vitamin E) and melatonin, suggesting the involvement of NMDAR activation and reactive species in these effects [68].

Other evidence revealed that MMA affects the glutamatergic neurotransmission in vivo. It was seen that the intrastriatal administration of MMA caused rotational behavior and clonic convulsions in rats, which were prevented by MK-801 [69]. Taken together, these studies strongly indicate that disturbances of glutamatergic neurotransmission, especially involving NMDAR overactivation, by MMA and PA underlie deleterious effects in the brain in MMAemia and PAemia.

### Disorders of Branched-Chain Amino Acid Metabolism

#### Maple Syrup Urine Disease

Maple syrup urine disease (MSUD) is caused by deficient activity of the branched-chain ketoacid dehydrogenase complex, blocking the metabolism of branched-chain keto acids (BCKA) and amino acids (BCAA). As a consequence, patients accumulate the α-keto-acids α-ketoisocaproic acid (KIC), α-keto-methylvaleric acid and α-ketoisovaleric acid and their respective amino acids leucine (Leu), isoleucine and valine [70, 71]. Clinical manifestations include episodes of vomiting, generalized convulsions, coma and respiratory distress, as well as progressive psychomotor delay, mental retardation and ataxia. Neuropathological findings generally reveal hypomyelination and cytotoxic intramyelinic sheath edema in the basal ganglia, cerebral cortex, cerebellum, periventricular white matter and brainstem, as well as cerebral atrophy [72, 73]. Treatment is based on a low-protein diet and a semi-synthetic formula low in BCAA supplemented with essential amino acids, vitamins and minerals [74].

Mounting evidence from humans and animal models supports an important role for accumulated metabolites, particularly leucine and α-KIC, in the pathogenesis of brain injury in MSUD [75]. A recent report showed that chronic administration of BCAA induced oxidative stress and inflammation, altered acetylcholine levels, and impaired memory in rats. It was also revealed that the NMDAR antagonist memantine mitigated these alterations [76], suggesting a possible role of a disturbed glutamatergic system in the brain damage observed in MSUD. Interestingly, memantine has been shown to enhance oligodendrocyte development and survival, as well as oligodendrocyte progenitor cell function, in experimental animal models of demyelination and neonatal brain injury [77]. Therefore, it is conceivable that memantine elicits positive effects in the MSUD rat model by reducing brain injury [76].

### Disorders of Glycine Metabolism

#### Nonketotic Hyperglycinemia (NKH)

Glycine cleavage system (GCS) is a mitochondrial enzyme complex composed of four proteins: the P-protein (glycine decarboxylase), T-protein (tetrahydrofolate aminomethyltransferase), H-protein (lipoic acid-containing carrier), and L-protein (lipoamide dehydrogenase). Mutations in the genes encoding glycine decarboxylase and aminomethyltransferase are the most common causes of nonketotic hyperglycinemia (NKH), a severe genetic disorder characterized by high accumulation of glycine (Gly) in body fluids and tissues of patients [78].

Most affected individuals present with the classic form of the disease that is manifested in the neonatal period with lethargy, coma, seizures and apnea, often leading to early death [79]. Brain MRI findings include progressive cortical atrophy, putamen abnormalities, and abnormal myelination in the white matter [79]. Treatment consisting of sodium benzoate and dextromethorphan is poorly effective [80, 81].

As glycine functions as a co-agonist of the NMDAR [82], the enhanced activation of this receptor, leading to excitotoxicity, has been proposed as an important mechanism underlying the pathophysiology of NKH [79, 83]. Other mechanisms proposed include disturbances in serine metabolism. Although glycine may lead to the formation of serine and subsequently D-serine, another NMDAR co-agonist, the levels of this amino acid are normal or low in NKH patients, suggesting that the levels of 5,10-methylene-THF, the cofactor for serine synthesis, are reduced [84, 85].

As NKH patients have high concentrations of this amino acid in the brain [79], several studies evaluated the toxicity of Gly, particularly related to the involvement of NMDAR in NKH pathophysiology. It was revealed that the intracerebral administration of Gly induced lipid peroxidation and disturbed mitochondrial bioenergetics in the cerebral cortex and striatum of young rats, which was prevented by the pre-treatment with MK-801 [86, 87]. Another in vitro work demonstrated that MK-801 normalized the decreased GSH levels caused by Gly in rat cerebellum [88].

It was also shown that intracerebroventricular injection of Gly in young rats impaired p38, ERK and JNK MAPK pathways, and reduced Tau phosphorylation and synaptophysin levels in the cerebral cortex and striatum of these animals, indicating neuronal damage [89]. Furthermore, and importantly, MK-801 pre-treatment attenuated the altered striatal p38 MAPK pathway, which is possibly involved in Gly-induced cell damage. Another work revealed that Gly decreased the levels of the NMDAR subunit 1 (NR1) in the cortex and increased the astrocyte glutamate transporter GLAST in the striatum of neonate rats, and induced oligodendrocyte and neuronal damage [90].

### Disorders of Glutamate/Glutamine and Aspartate/Asparagine Metabolism

#### Aspartoacylase Deficiency

Aspartoacylase (ASPA) deficiency, also known as Canavan disease, is caused by mutations in the *aspartoacylase* gene and leads to a high accumulation of N-acetylaspartate (NAA) and its derivative N-acetyl-aspartyl-glutamate (NAAG) in the brain [91]. Patients present with severe intellectual disability, hypotonia, macrocephaly and generalized tonic and clonic type seizures. MRI reveals generalized leukodystrophy, characterized by diffusechanges in the subcortical white matter and cortical regions, as well as involvement of the brainstem and cerebellum. Globus pallidus abnormalities are also a common finding [92]. There is currently no specific treatment for this disorder [92].

 In vitro effects of NAA and NAAG on glutamatergic neurotransmission were reported in cell cultures and rodent models. Electrophysiological studies showed that NAAG evoked an inward membrane current in cerebellar oligodendrocytes, which was reduced by blocking NMDAR but not by blocking metabotropic glutamate receptors [93]. It was also seen that NAA and NAAG evoked a current in cerebellum granule cells and that the NMDA receptor blocker D-AP5 blocked the NAAG-evoked current both in oligodendrocytes and in granule cells. Furthermore, NAAG also induced a rise in [Ca^2+^]i only in neurons but not in oligodendrocytes [93]. These data suggest that NAAG mainly activates neuronal NMDAR and that the current evoked by NAAG in oligodendrocytes reflects a consequence of the activation of neuronal NMDAR.

The so-called tremor rat is a mutant animal that exhibits a genomic deletion of the *aspartoacylase* gene with absence-like seizures, convulsions, and vacuole formation in the CNS [94, 95]. It was shown that the increase in NAA levels in the CNS of the tremor rats occurs in parallel with the severity of vacuole formation [96]. Consistent with this, the same study revealed that intracerebroventricular administration of NAA to rats induced abnormal EEG with absence-like seizures accompanied by sudden immobility and staring, followed by convulsive seizures in a dose-dependent manner [96].

Additionally, NAA was shown to induce an inward current through metabotropic glutamate receptors, which are coupled to the G protein, leading to excitation in hippocampal neurons and contributing to the occurrence of epileptic seizures [97]. It was also reported that intracerebroventricular injection of NAA induced prolonged seizures in rats, indicating the involvement of glutamate receptors [98].

#### Glutamine Synthetase Deficiency

Glutamine synthetase (GS) deficiency is a rare recessive disorder characterized by decreased glutamine (Gln) levels in body fluids and associated with chronic hyperammonemia [99]. Three unrelated patients have been reported so far [100]. Clinical manifestations include severe neonatal encephalopathy and brain malformations such as general atrophy, abnormal gyration, and white matter lesions [100].

The pathophysiology of GS deficiency is poorly understood. However, studies have suggested that the reduction of Gln levels may disturb the Glu/Gln cycle between astrocytes and neurons, thus affecting Glu concentrations in the brain and potentially altering glutamatergic neurotransmission [100, 101].

## Disorders of Ketone Body Metabolism

### 3-Hydroxy-3-methylglutaryl-CoA Lyase Deficiency

3-Hydroxy-3-methylglutaryl-CoA lyase (HL) deficiency is an autosomal recessive IEM characterized by predominant tissue accumulation and high urinary excretion of 3-hydroxy-3-methylglutarate (HMG), apart from 3-methylglutarate (MGA), 3-methylglutaconate, 3-hydroxyisovalerate and 3-methylcrotonylglycine, which also accumulate in this disease. The onset of symptoms usually happens in the first year of life and worsens during fasting or other catabolic events [102–104]. Affected patients present with metabolic acidosis, hypoketotic hypoglycemia, hypotonia, lethargy, convulsions and coma. White matter abnormalities accompanied by abnormalities of the basal ganglia and cerebral atrophy are commonly observed [102]. Treatment is based on a low-protein diet, L-carnitine therapy with a high-carbohydrate supplement [102, 105].

 In vivo and in vitro studies carried out in the brain of rats have demonstrated the role of NMDAR in the pathophysiology of HL deficiency. Intrastriatal administration of HMG in rat brain caused lipid and protein oxidative damage as well as increased reactive oxygen and nitrogen species. MK-801 pre-treatment prevented these effects. Moreover, HMG decreased GSH concentrations and altered the activities of antioxidant enzymes, which were also mitigated by MK-801 [106], indicating a role for NMDAR in these effects.

It was also revealed that MGA, another metabolite accumulated in HL deficiency, increased lipid peroxidation and impaired mitochondrial function in vitro in synaptosomal and mitochondrial fractions prepared from rat brain. The cannabinoid receptor agonist WIN55,212-2 and the NMDAR antagonist kynurenic acid mitigated these effects [108. In this context, it was found that activation of cannabinoid receptors reduces presynaptic Glu release through a direct action on the calcium channel of NMDAR. Furthermore, a lower dose of MGA combined with the NMDAR [107] agonist QUIN similarly induced oxidative stress and compromised the mitochondrial function, which were abolished by the NMDAR antagonist kynurenic acid [108]. These data suggest a glutamatergic component in the synergic effects induced by MGA and QUIN.

On the other hand, MK-801 and the selective antagonist of the extrasynaptic NMDAR-containing NR2B subunit ifenprodil mitigated HMG-induced hypophosphorylation of GFAP and the neurofilament subunits NFL, NFM, and NFH in the striatum of rats. In line with this, the same study demonstrated that Ca^2+^ buffering and the inhibition of nitric oxide synthase also attenuated these effects, highlighting the relevance of NMDA-mediated protein phosphorylation. Downregulation of PKA and JNK/MAPK active subunits was also seen, indicating that these kinases have an important role in the NMDAR-mediated effects [109].

## Other Disorders of the Intermediary Metabolism

D-2-Hydroxyglutaric aciduria (D-2-HGA) and L-2-hydroxyglutaric aciduria (L-2-HGA) are neurometabolic disorders biochemically characterized by the accumulation of D-2-hydroxyglutaric acid (D-2-HG) and L-2-hydroxyglutaric acid (L-2-HG), respectively, predominantly in the brain and biological fluids of the affected patients [110]. Two variants of D-2-HGA have been found: D-2-HGA type I, which is caused by mutations in the *D-2-hydroxyglutarate dehydrogenase* gene (*D2HGDH*), and D-2-HGA type II, caused by gain-of-function mutations in the *isocitrate dehydrogenase II* (*IDH2*) gene. Both forms of D-2-HGA can trigger neonatal or early childhood epileptic encephalopathy, with hypotonia, developmental delay, seizures, and even cardiomyopathy, although D-2-HGA type II is usually more severe [8, 110]. Neuroimaging analysis demonstrates enlargement of the lateral ventricles and frontal subarachnoid spaces, subdural effusions, subependymal pseudocysts, signs of delayed cerebral maturation and white matter abnormalities [110].

L-2-Hydroxyglutaric aciduria (L-2-HGA) is caused by mutations in the gene encoding L-2-hydroxyglutarate dehydrogenase (LHGDH), leading to the accumulation of L-2-HG in the brain and biological fluids, which is the biochemical hallmark of L-2-HGA [111, 112]. L-2-HGA exclusively affects the CNS, although the *LHGDH* gene is expressed in multiple tissues, such as skeletal muscle and testis, besides the brain [111]. Patients usually manifest with psychomotor regression, variable motor alterations, and epilepsy, which appear during infancy and early childhood, gradually evolving to cerebellar ataxia, moderate to severe cognitive impairment, and pyramidal and extrapyramidal signs [113, 114]. MRI findings reveal subcortical and deep white matter hyperintensities involving the dentate nucleus and basal ganglia, progressing to deeper layers with a centripetal extension of the white matter lesions [115, 116].

 In vivo animal studies have shown that glutamatergic neurotransmission is disturbed in D-2-HGA and L-2-HGA. It was verified that MK-801 prevented reactive oxygen and nitrogen species generation, antioxidant system disturbances and lipid and protein oxidative damage provoked by intracerebral administration of D-2-HG and L-2-HG to rats [117, 118]. Other in vitro studies revealed that L-2-HG increases Glu uptake in rat brain synaptosomes [119] and that D-2-HG stimulates NMDAR-mediated excitotoxicity in neuronal chick and rat cultures [120].

## Concluding Remarks and Perspectives

We reviewed here many in vivo and in vitro experimental studies, implying disturbances of the glutamatergic system caused by metabolites accumulating in various IEMs, which are clinically characterized by convulsions associated with neurodegeneration. The presented data evidenced that excitotoxicity may represent an important underlying pathomechanism of brain damage in these disorders. Some studies revealed direct or indirect effects of the accumulating metabolites disturbing critical parameters of the glutamatergic system. Other works utilizing chemical and genetic models of IEMs demonstrated that NMDAR antagonists mitigated cellular events linked to excitotoxic damage in vitro and in vivo. In this particular, deleterious mechanisms of brain damage often associated with and usually consequent to excitotoxicity, such as oxidative stress and bioenergetic disruption elicited by the accumulated metabolites, were prevented by blocking the overstimulation of NMDAR that leads to a high influx of calcium into the cells and are followed by increased production of ROS and impairment of electron transport chain (ETC) [121]. Noteworthy, the inhibition of ETC complexes caused by the mitochondrial accumulation of calcium may also be a source for elevated ROS levels.

On the other hand, NMDAR antagonists have not been used in the treatment of IEMs. One exception is NKH, whose management initially included the administration of the partial antagonists dextromethorphan or ketamine to reduce seizures, but this has been a matter of debate owing to the lack of clear clinical evidence of therapeutic benefits and to the potential adverse effects of these drugs [122]. Controlled clinical studies with dextromethorphan, ketamine or other NMDAR-type glutamatergic receptor inhibitors have never been done. These observations do not rule out that partial NMDAR modulators, also including memantine or felbamate, as well as neurosteroids, and mGluR ligands, should be explored as potential therapies to improve the outcome of patients with IEMs, after well-controlled clinical trials with validated endpoints that should be developed to allow a clinical evidence base for their use. However, particularly in the case of rare disorders, these new approaches should be applied to many patients through collaboration between physicians in multiple centers.

In summary, the data reviewed here provide important insights into the pathophysiology of some IEMs. However, additional research, especially in cell models (e.g., iPS cells and differentiated neurons from patients, or brain organoids), is needed to confirm whether excitotoxicity is involved in the pathogenesis of the brain damage in these disorders. If this is the case, it is necessary to decipher the precise signaling pathways involved before adequate treatment is implemented.

## Data Availability

No datasets were generated or analysed during the current study.
